# Parasitic plants: physiology, development, signaling, and ecosystem interactions

**DOI:** 10.1093/plphys/kiab055

**Published:** 2021-04-21

**Authors:** Harro Bouwmeester, Neelima Sinha, Julie Scholes

**Affiliations:** 1 Swammerdam Institute for Life Sciences, University of Amsterdam, PO Box 94215, Amsterdam, 1090GE, The Netherlands; 2 Department of Plant Biology, University of California, Davis, CA 95616, USA; 3 Department of Animal and Plant Sciences, University of Sheffield, Sheffield, S10 2TN, UK

Parasitic plants connect to the vasculature of a host plant and take part or all of the water, nutrients, and assimilates they need to complete their life cycle. Parasitic plants represent a unique model for the evolution of intra-kingdom parasitism with intriguing research questions such as how plants were able to evolve the ability to parasitize other plants. This parasitic lifestyle required the evolution of host detection, host attachment, host exploitation, and host defense suppression strategies. The elucidation of these strategies and the underlying mechanisms has been greatly facilitated by the advent of model species, molecular and genomic tools, and omics approaches such as transcriptomics and comparative genomics and this has launched parasitic plant research into the 21st century. The scientific data generated are helping us to gradually unravel how parasitism in plants has evolved. However, parasitic plants are not just an interesting basic research model. Due to their parasitic lifestyle, many parasitic plant species represent important agricultural weeds, such as the witchweeds, broomrapes, dodders, and mistletoes. Fundamental knowledge of the mechanisms underlying parasitism will also contribute to new approaches to create resistance in crops and develop new control measures for this important agricultural problem.

This Focus Issue on Parasitic Plants addresses some of the most important advances and new landmarks in the field. In addition to commissioned updates by experts in their respective topics, several research articles highlight recent accomplishments in these areas.

The name “parasitic plant” illustrates that these plants grow at the expense of their hosts and can cause problems in agriculture, as will be further discussed below. But parasitic plants are also an essential component of biodiverse ecosystems. In two updates, Casadesús and Munné-Bosch (2021) and Těšitel et al. (2021) describe the role of parasitic plants in natural ecosystems. Casadesús and Munné-Bosch (2021) highlight the role of holoparasitic plant–host interactions (such as those between *Cytinus hypocistis* and various shrubs of the genus Cistus) in shaping natural Mediterranean ecosystems. Těšitel et al. (2021) discuss how parasitic plants can also positively influence the growth and/or reproductive output of their host or other, nonhost, organisms and that they can have positive effects on community structure and ecosystem processes. Těšitel et al. (2021) also review the roles that parasitic plants may have as food or medicinal resources or for aesthetic and cultural purposes.

During the early days of genomics, it was assumed that parasitic plants must have acquired pathogenicity genes through horizontal gene transfer. Surprisingly, in the last decade or so it also became clear that pathogenicity evolved from extant physiological processes also present in nonparasitic plant species. This Focus Issue contains several examples of the recruitment by parasitic plants of genes involved in these extant physiological processes, to facilitate aspects of the parasitic process. A prime example is the coordination of the parasitic plant lifecycle with that of the host, aided by a number of host detection/host response mechanisms that have evolved in parasitic plant species. In the update by Brun et al. (2021), the molecular mechanisms underlying two of these—host-dependent seed germination and haustorium formation—are reviewed. The key process of germination in parasitic plants in response to molecules exuded by their host is also discussed in the updates by Bouwmeester et al. (2021) and Nelson (2021) and in the research article by Wang et al. (2021). Especially in the Orobanchaceae, germination in response to chemical host cues—particularly the strigolactones—is a striking example of the evolution of a host detection mechanism. Intriguingly, strigolactones have been shown to be essential host signaling molecules, making them reliable cues for the parasites (Bouwmeester et al., 2021). Also, the evolution of a perception mechanism by parasitic plants for the germination stimulants, especially strigolactones, by recruiting homologs of a receptor KARRIKIN INSENSITIVE 2 (KAI2)/HYPOSENSITIVE TO LIGHT (HTL) and modification of their ligand-binding cavities to allow binding of strigolactones, and their possible role in host selection is highly intriguing (Bouwmeester et al., 2021; Brun et al., 2021; Nelson, 2021). In their research article, Wang et al. (2021) study one of these germination stimulant receptors, ShHTL7, in more detail and demonstrate the key role of the strigolactone-induced interaction of ShHTL7 with F-Box protein MORE AXILLARY GROWTH 2 (MAX2) and repressor of gene expression, SUPPRESSOR OF MORE AXILLARY GROWTH2 1 (SMAX1) and the effect of this complex formation on the sensitivity to strigolactones.

Just as for regulation of germination, parasitic plants seem to have hijacked extant physiological processes for the attachment to and penetration of host plant tissues by the haustorium. Haustorium formation is an intriguing process, divided into three phases by Brun et al. (2021): (1) attachment to host tissue, (2) penetration of the host, and (3) connection to the host vasculature. The authors review the current knowledge on the role of haustorium inducing factors (phenolic molecules secreted by the host that induce haustorium formation), plant hormones such as cytokinin and auxin, cell-wall degrading enzymes produced by the parasite, and avoiding host detection in haustorium formation.

In two research articles by Masumoto et al. (2021) and Ogawa et al. (2021), new insights into these processes are presented. Masumoto et al. (2021) report a three-dimensional reconstruction of the haustorium of the obligate parasite *Striga hermonthica* and the facultative parasite *Phtheirospermum japonicum*. Using this reconstruction in combination with field-emission scanning electron microscopy they reveal the spatial arrangements of multiple cell types inside haustoria and their interaction with the host root, and highlight differences between the two parasites, particularly with regard to the xylem connection with the host.

Ogawa et al. (2021) investigated differential gene expression in the intrusive cells of *P. japonicum* and used promoter analyses to characterize a number of the differentially expressed genes they identified. They pay particular attention to four subtilisin-like serine proteases, which, upon overexpression, inhibited both intrusive cell and xylem bridge development, suggesting they play an important role in haustorium development in *P. japonicum*.

The haustorium plays an essential role in the transfer of assimilates, amino acids, and nutrients from the host to the parasite. In a research article, Zhang et al. (2021) show that dodder transfers systemic signals, including long-distance mobile mRNAs, with information on the nitrogen status, from one host to another. They demonstrate that these systemic signals are able to regulate large transcriptome and DNA methylome changes in the recipient hosts. Intriguingly, in the case of host species soybean and cucumber, signals produced by N-starved soybean, induced increased N uptake activity in cucumber.

Parasitic plants have not only recruited extant physiological processes, to support their parasitic lifestyle, they have also lost certain functions. An intriguing example of this is the loss of photosynthesis, and many of the genes required for that process, by holoparsitic species such as the broomrapes. Intriguingly, in their research article, Gua et al. (2021) demonstrate that the biosynthesis of phylloquinone, involved in photosystem I electron transport and disulfide bridge formation of photosystem II subunits, is retained by the nonphotosynthetic holoparasite *Phelipanche aegyptiaca*. The authors suggest that nonphotosynthetic holoparasites exploit alternative targeting of phylloquinone for transmembrane redox signaling associated with parasitism.

Research on parasitic plants has greatly benefited from the ever growing genomics toolkit. To support comparative genomics work on parasitic plants, Kösters et al. (2021) in a Breakthrough Technologies paper describe a new tool, the Web Application for the Research of Parasitic Plants (WARPP). WARPP should facilitate international efforts by providing a central hub of curated evolutionary, ecological, and genetic data on parasitic plants. The tool includes a genome browser for parasitic plant genomes, an orthogroup summary table, and a tool to explore ancestral traits. WARPP hopes to benefit from contributions from the scientific community, see https://parasiticplants.app.

The advances in our knowledge underlying the relationship between hosts and parasitic plants, have greatly improved our understanding of the evolution of plant parasitism. Moreover, it is facilitating the development of more effective control measures in cases where these parasitic plants have developed into agricultural weeds. The threat imposed to agriculture by these weeds is nicely illustrated in the research article by Masanga et al. (2021). They reveal the presence of field dodder (*Cuscuta campestris*) and *C. kilimanjari* (both either naturalized or endemic to East Africa), and for the first time in continental Africa, the presence of the giant dodder (*C. reflexa*) a south Asian species. These parasites have a wide host range, parasitizing species across 13 angiosperm orders. They studied the potential risk of *C. reflexa* expanding its host range to important woody cash crops such as tea, coffee, and mango and revealed successful parasitism, following haustorial formation and vascular bundle connections in all three crops. Modeling further supported the threat, by predicting high habitat suitability for all three *Cuscuta* species across major tea- and coffee-growing regions of Eastern Africa.

In addition to the large agricultural problems with parasitic plants in the African continent, the Mediterranean is heavily plagued by broomrape problems in a multitude of crops. In their research article, Casadesús and Munné-Bosch (2021) discuss the impact of these broomrapes infecting economically important dicotyledonous crops in Mediterranean agroecosystems.

In an update, Jamil et al. (2021) describe how our growing knowledge of the mechanisms underlying plant parasitism can be used to improve their control, both through (modern) breeding and through smart agricultural practices and agro-chemistry. In order to be able to introduce resistance into crops, knowledge of the underlying mechanisms is required. Intriguingly, most hosts are unable to detect an infection by plant parasites or are unable to fend them off. In their update, Albert et al. (2021) review examples of hosts that have evolved defense strategies to avoid infection or protect themselves actively post-attack often leading to full or partial resistance. They discuss the current state of our understanding of the defense strategies to plant parasitism used by host plants with emphasis on the active molecular resistance mechanisms and outline the perspectives and the potential of future studies needed to develop and breed-resistant crops.

We hope that the contents of this focus issue will draw attention to the importance of parasitic plants in natural and agricultural ecosystems. Using physiological, biochemical, structural, developmental, and genomics tools the authors have shed light on fundamental processes involved in the parasitism process and the host response to parasitism.

## Notes

The author responsible for distribution of materials integral to the findings presented in this article in accordance with the policy described in the Instructions for Authors (https://academic.oup.com/plphys/pages/general-instructions) is: Harro Bouwmeester (H.J.Bouwmeester@uva.nl).
